# The collective effect of finite-sized inhomogeneities on the spatial spread of populations in two dimensions

**DOI:** 10.1098/rsif.2021.0579

**Published:** 2021-10-20

**Authors:** Wolfram Möbius, Francesca Tesser, Kim M. J. Alards, Roberto Benzi, David R. Nelson, Federico Toschi

**Affiliations:** ^1^ Living Systems Institute, University of Exeter, Exeter, UK; ^2^ Physics and Astronomy, College of Engineering, Mathematics and Physical Sciences, University of Exeter, Exeter, UK; ^3^ Department of Applied Physics, Technische Universiteit Eindhoven, Eindhoven, The Netherlands; ^4^ Department of Physics, Harvard University, Cambridge, MA, USA; ^5^ Department of Molecular and Cellular Biology, Harvard University, Cambridge, MA, USA; ^6^ PMMH, ESPCI Paris—PSL, Paris, France; ^7^ Universitá di Roma ‘Tor Vergata’ and INFN, Rome, Italy; ^8^ Istituto per le Applicazioni del Calcolo, Consiglio Nazionale delle Ricerche, Rome, Italy

**Keywords:** front propagation, range expansion, Fermat’s principle of least time, heterogeneous environment, individual-based simulation

## Abstract

The dynamics of a population expanding into unoccupied habitat has been primarily studied for situations in which growth and dispersal parameters are uniform in space or vary in one dimension. Here, we study the influence of finite-sized individual inhomogeneities and their collective effect on front speed if randomly placed in a two-dimensional habitat. We use an individual-based model to investigate the front dynamics for a region in which dispersal or growth of individuals is reduced to zero (obstacles) or increased above the background (hotspots), respectively. In a regime where front dynamics is determined by a local front speed only, a principle of least time can be employed to predict front speed and shape. The resulting analytical solutions motivate an event-based algorithm illustrating the effects of several obstacles or hotspots. We finally apply the principle of least time to large heterogeneous environments by solving the Eikonal equation numerically. Obstacles lead to a slow-down that is dominated by the number density and width of obstacles, but not by their precise shape. Hotspots result in a speed-up, which we characterize as function of hotspot strength and density. Our findings emphasize the importance of taking the dimensionality of the environment into account.

## 1. Introduction

Populations spread into yet-unoccupied habitats on a wide range of length and time scales. Prominent examples are the spread of invasive plants on large spatial scales and the growth of microbial populations on small spatial scales. Despite being so different at first sight, all these population expansions are driven by two processes, population growth and active or passive dispersal [1–3]. While the former drives overall growth of the population, i.e. the number of individuals, the latter is necessary for the population to spread into new habitat.

**Figure 1.**
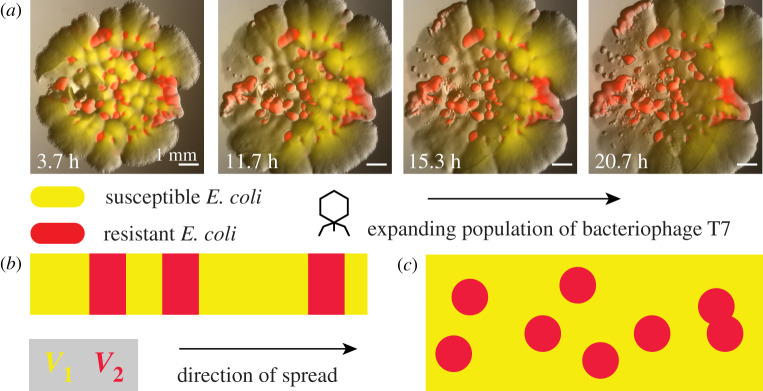
(*a*) Experimental realization of a population front encountering a heterogeneous environment. A population of bacteriophage T7 (dark area) is expanding on a lawn of *E. coli*, where yellow areas represent patches of bacteria which can be infected by the bacteriophage (i.e. in which the population front can expand), while red areas represent patches of *E. coli* which are known to be resistant (see [4] for a description of the experiment and additional information). (*b*) Sketch of an effectively one-dimensional heterogeneous environment where red and yellow patches differ in their support for population expansion by allowing different expansion speeds, *v*_1_ and *v*_2_, respectively. (*c*) Like (*b*), but for a two-dimensional environment.

The environment encountered by these populations is often heterogeneous, i.e. the growth or dispersal processes may vary locally. An example is displayed in figure 1*a*: a population of a bacterial virus is expanding in a heterogeneous environment consisting of two types of bacteria. A region of bacteria which supports growth of the virus population (indicated in yellow, by use of yellow fluorescent proteins inside bacteria) is interspersed with regions of bacteria that do not support growth of the phage population (indicated in red) [4].

Much work has focused on heterogeneous one-dimensional environments such as depicted in figure 1*b*, where yellow and red indicate two different kinds of patches with specified population growth and dispersal (e.g. [5–7] and references therein). For example, considering linear periodic habitats, Shigesada *et al.* [8] studied invasion conditions of migrating species and the resulting periodic travelling waves. Limiting oneself to one-dimensional space not only simplifies the theoretical treatment but also describes expansions in linear habitats such as along coastlines, watercourses or transportation networks.

Care has to be taken when generalizing the results from studies of one-dimensional environments to higher dimensions. This is because results from linear habitats generally cannot be easily transferred: consider figure 1*b* with a scenario where the red patches slow down an invasion so it almost comes to a halt. Due to the alternating position of red and yellow patches, these isolated red patches thus have a dramatic influence on the overall invasion process. The situation is different in two dimensions if the red patches are of finite size, yet isolated, as in figure 1*c*. In this case, as we will show, they affect the overall invasion process only marginally for low to intermediate densities, because invading populations can envelope finite-sized obstacles. Two-dimensional habitats are realized at the surfaces of solid substrates or liquids. Accordingly, our findings may find applications in the field of landscape ecology of invasive spread [9], complementing existing simulation-based work [10–13]. In addition, effectively two-dimensional populations can be found embedded in other environments, such as thin phytoplankton layers in the ocean [14].

We here consider two different types of inhomogeneities. They may be associated with a population growth rate that is different from that of the embedding environment or may be regions within which dispersal of individuals differs. We find that significant progress can be made in a regime where the locally varying growth and dispersal properties result in a well-defined locally varying front speed that is independent of front speed at other locations or times. This regime has an analogy in geometrical optics where the refractive index and thus the speed of light vary locally. In consequence, our findings for front propagation in the presence of finite-sized inhomogeneities may be relevant for a range of propagation phenomena that share the trait of locally varying front speed, but not necessarily the underlying mechanism for front propagation: the spread of bacteriophage on a bacterial lawn (figure 1*a*), invasive brain tumours for which it is essential to differentiate tumour cell motility in white and grey matter [15], the propagation of flame fronts [16], and autocatalytic reactions in porous media [17].

Note that locally varying dispersal does not necessarily mean that individuals move differently. Under certain circumstances, e.g. slow reaction or small-scale turbulence (thickness of the front much broader than the scale of turbulent eddies), the effect of turbulent background flows can also be described by an effective total diffusivity [18]. Thus, the example of turbulent patches with a position-dependent effective diffusivity broadens the scenarios we consider.

Our findings build on recent studies that considered isolated obstacles to two-dimensional population expansions [4] and expansions over curved surfaces [19], but expands beyond them: we here consider the converse of obstacles to invasions and characterize their consequences. Furthermore, instead of focusing on individual inhomogeneities, we investigate a whole range of environments, from those with isolated inhomogeneities to environments where features are so abundant that they almost fill up the two-dimensional space. The features considered are of finite size and randomly distributed, complementary to work focusing on purely random, two-dimensional periodic and fractal-based environments [20–22].

## 2. Individual-based simulations

An expanding population can be described at different levels of detail or coarsening. We first consider an individual-based scheme which allows us to take discreteness and random fluctuations into account directly. Individuals in the population can undergo growth and dispersal [23], whereby the growth process includes both birth and death of individuals.

**Figure 2.**
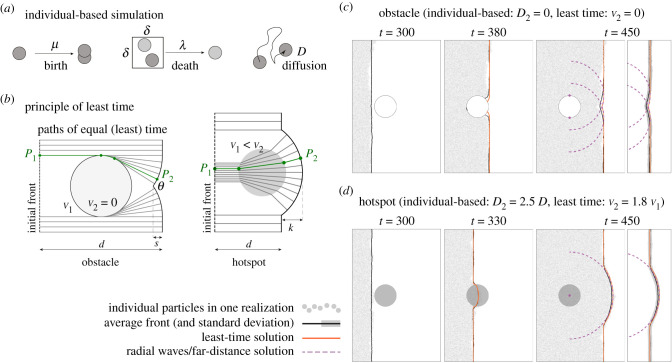
(*a*) Sketch of the individual-based model with birth, death and diffusion. Duplication of individuals occurs with rate *μ*, death by competition within a squared cell of size *δ*^2^ with two individuals at rate *λ*. *D* is the diffusion coefficient. (*b*) Least-time consideration for an obstacle (left, [4]) and a hotspot (right, electronic supplementary material, appendix S2). There, the green line is one example for a path of least time from point *P*_2_ back to the initial condition, which is reached at point *P*_1_. The other grey lines represent paths of virtual markers travelling from left to right in the same amount of time. (*c*) Results of the individual-based simulation with an obstacle (white circle) with radius *R* = 50 and *D*_2_ = 0 (grey dots), overlaid by the average front obtained from multiple realizations (black line, outside the obstacle), the least-time solution (orange line), and the far-distance solution (radial waves, purple dashed lines); see also electronic supplementary material, video S1, [4]. Right-most panel indicates standard deviation to average front instead of individual particles. (*d*) Similar to (*c*), but the obstacle is replaced by a hotspot (grey circle) with radius *R* = 50 and *D*_2_ = 2.5*D* (electronic supplementary material, video S2).

Birth is a duplication of an existing individual without change of position that occurs at rate *μ*. Death is disappearance of an individual through competition and is dependent on the amount of neighbouring individuals. The two-dimensional domain is subdivided into fixed square interaction cells of area *δ*^2^. An individual disappears at rate *λ* · *n* when *n* *other* individuals are present in the same lattice cell. Thereby, *λ* is a rate independent of *n*. The birth and death processes can be described by the binary interactions sketched in figure 2*a* and can be summarized as

2.1 $$X\overset{\mu }{\to }X+X\;X+X^{\prime }\;(\mathrm{inside}\;\delta^{2})\overset{\lambda }{\to }X^{\prime }.$$

This choice of rules is also known as birth-coagulation process as disappearance occurs through coagulation [24].

In addition to birth and death, individuals are subject to dispersal in the form of a random walk, i.e. they diffuse in a two-dimensional continuous habitat with diffusion coefficient *D*, as depicted in figure 2*a*. This diffusive motion, together with the birth–death process, allows one to interpret the individual-based scheme as a discretized reaction–diffusion scheme (electronic supplementary material, appendix S1).

All individual-based simulations are performed with a domain size of 1000 × 1000, an interaction range of *δ* = 1, a diffusion coefficient of *D* = 1 and birth and death rates of *μ* = 1 and *λ* = 1, respectively, unless specified otherwise.

When a band of individuals is set as initial condition, the system invades the empty space with a fluctuating front propagating at an average constant speed controlled by the microscopic parameters and the associated level of demographic noise, which is larger for smaller density [24,25].

## 3. Single circular obstacles and hotspots

Inhomogeneities within which the microscopic parameters differ from their values outside are expected to shape the dynamics of the front. We refer to a patch that slows down or blocks the front as an ‘obstacle’ and to a region that can be invaded faster than the surroundings as a ‘hotspot’.

First, we study the effect of one single circular impermeable obstacle, realized by a locally vanishing diffusion coefficient, *D*_2_ = 0. Figure 2*c* shows a time series of a single realization of an individual-based simulation as well as the average front obtained from many realizations (see electronic supplementary material, video S1 for all frames and electronic supplementary material, appendix S1 on how the front is determined). We observe that right after the front has passed the obstacle, a part of the front lags behind, resulting in a kink that then heals. This behaviour is in qualitative agreement with the observations of [4], where a ‘constant speed model’ was used to describe front shape when a population front encounters an obstacle. In this model, the front results from a collection of points that have the same distance to the initial front when taking into account the impermeability of the obstacle as sketched in figure 2*b*. The green line gives one example of a shortest path or ‘path of least time’ between a point at the front and any point at the initial condition. The total front is constructed by finding all points that have the same distance to the initial front; see [4] for details. In figure 2*c*, we show this least-time front for a complete propagation around an obstacle (orange line). We observe that this construction recovers the average shape of the front from the simulations, including the kink, very well. The individual front is slightly lagging behind however as observed before [4]. Far away from the obstacle the front is well described by the envelope of two radial waves (dashed purple lines in figure 2*c*), initiated from the two vertical extremes of the obstacle and travelling with constant speed as will be discussed below.

The reverse situation of a ‘hotspot’ can be achieved by setting the diffusion coefficient of individuals larger inside the inhomogeneity than in its surrounding. Figure 2*d* shows the results of simulations where the diffusion coefficient inside the circular patch, *D*_2_, is 2.5 times larger than outside (see electronic supplementary material, video S2 for all frames and electronic supplementary material, appendix S1 for details). The population expands faster within the hotspot, and a bulge forms to the right of the hotspot. The front dynamics can be described using a least-time consideration that assumes two different propagation speeds, *v*_2_ and *v*_1_ < *v*_2_, inside and outside the hotspot, respectively; see figure 2*b*. The front consists of the set of points whose paths back to initial condition are traversed in the same amount of minimal time (compared to alternative paths), in analogy to ‘Fermat’s principle of least time’ from classical optics [26]. Using Snell’s Law, which can be derived from ‘Fermat’s principle of least time’, the resulting front dynamics can be obtained analytically (electronic supplementary material, appendix S2, figure S1). With *v*_2_ ≈ 1.8*v*_1_ (estimated from simulations of homogeneous systems), the resulting solution approximately captures the shape observed in the individual simulations (figure 2*d*, orange line). A combination of the planar front and a radial wave (dashed purple line in figure 2*d*), emitted from the centre of the hotspot describes front shape well far away from the hotspot, as will be explored below.

Overall, we find that a least-time description of front dynamics allows us to describe the dynamics of a population front encountering a single obstacle, within which diffusivity vanishes, or a single hotspot, a region where diffusivity is increased. Completely analogous observations are made when a population wave encounters a region with vanishing or increased birth rate (instead of diffusivity); see electronic supplementary material, figure S3.

## 4. Applicability of the least-time principle

Before applying the least-time approach to more complex shapes and heterogeneous environments, we briefly outline its range of validity. The validity of the least-time description relies on the possibility of replacing the dynamics of the whole population by an interface propagating orthogonal to itself with a locally defined speed, i.e. a speed that depends on location only and not on, for example, direction or front dynamics at earlier times.

Population fronts are characterized by a transition from an unstable to a stable state. At a specified location, this transition occurs over a finite time. Spatially, this transition presents itself as a finite steepness of the front which corresponds to a front width. Note that this is a different measure from front or interface roughness, which can also be referred to as width. The coarsened, least-time approach implicitly assumes vanishing width. For the least-time approach to be a good description of the full dynamics, widths of travelling fronts firstly need to be very small compared to length scales of the system, i.e. the typical size of obstacles and hotspots or spacings between them.

Secondly, transient regimes are expected when a population encounters a hotspot or leaves it behind as the front does not instantaneously change speed and steepness as it passes from one type of environment to another. These associated times are required to be negligible with respect to the time the front takes to pass through the hotspot. This condition is difficult to quantify, but can always be met by sufficiently large scales.

Thirdly, local front curvature is expected to have an effect on front speed in the underlying microscopic model [27] not reflected in the coarsened model where front speed is a purely local parameter. This effect can be important at the corner of an obstacle [4], at the entrance of a hotspot, or at the kinks of perturbed fronts. Although the least-time approach does not capture these subtleties, their relative effect is expected to be small for large features.

We stress that individual-based models are particularly suitable to the study of heterogeneous media, since the presence of a natural cut-off (due to the discreteness of the individuals) leads to a unique and stable front speed [28].

Finally, individual-based models are characterized by a natural roughness due to the stochastic nature of the growth process [29]. In this paper, we consider situations in which the size of the feature and the perturbation to the front by an obstacle or a hotspot are large compared to the typical scale of the roughness. For hotspots, this criterion depends not only on its size but also on its strength.

## 5. Obstacle width and hotspot length shape front at large distances

The least-time considerations can be used to uncover which aspects of an obstacle’s or hotspot’s shape dictate front shape far away from the feature. Figure 2*b* shows that the front in the shadow of the obstacle is associated with paths originating from an area around the obstacle’s maximum width. These paths are the shortest path back to the initial front (compare also [4]). Electronic supplementary material, figure S2A depicts the front further downstream, highlighting this observation. This suggests that (i) the exact shape of the obstacle does not matter for the front shape far downstream and that (ii) two radial fronts, each originating from the widest part of the obstacle, describe the solution for general obstacle shapes at large distances downstream.

To test these arguments, we determined the fronts numerically for more general shapes, employing the fact that the least-time consideration is equivalent to the Eikonal equation,

5.1 $$|\nabla T(\vec{x})|=1/v(\vec{x}),$$

which connects the arrival time $T(\vec{x})$ to the local front speed $v(\vec{x})$. Front shapes at different times are given by contours in the arrival time $T(\vec{x})$, which can be numerically obtained using the fast marching method [30,31].

**Figure 3.**
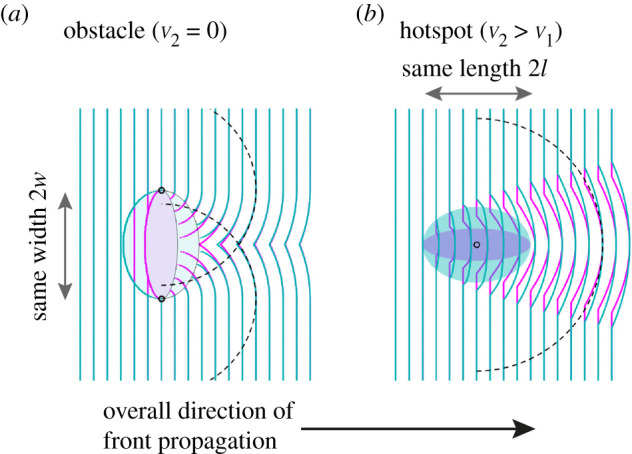
Front shape before and after encountering an obstacle and hotspot, respectively, with front speed *v*_1_ outside the feature and *v*_2_ inside. (*a*) Solutions of the Eikonal equation (magenta and cyan lines) at different times relative to elliptical obstacles (*v*_2_ = 0, magenta and cyan-shadowed ellipses) with equal widths but varying aspect ratio. Half-circles originating from the sides of the obstacle (dashed lines with origins marked by black circles) capture front shape downstream from the obstacle. (*b*) Numerical solutions relative to elliptical hotspots (*v*_2_/*v*_1_ = 1.2, magenta and cyan ellipses) with equal length but varying aspect ratio. A half circle originating at the centres of the hotspots with radius given by equation (5.3) describes the bulge in the front downstream from the hotspots.

We chose two different elliptical obstacles with the same width, but different lengths, and computed the front numerically as depicted in figure 3*a*. Indeed, we observe that obstacles with the same width perturb the front far away from the obstacle in the same manner. The far-distance solution, constituted by two half circles, matches the numerical solution very well. To illustrate that the effect is not limited to convex shapes, we repeated the computation for a tulip-shaped obstacle, see electronic supplementary material, figure S4A, and again observe very good agreement.

The healing of the kink induced by the obstacle can be quantified by the opening angle *θ* and the indent size *s* indicated in figure 2*b*. For large distances travelled since the obstacle was encountered we obtain:

5.2 $$\theta \approx \pi -\frac{2w}{d},\;s\approx \frac{w^{2}}{2d},$$

where *w* is the half-width of the obstacle (equal to radius for circular obstacle) and *d* is the distance travelled since the front has passed the point of maximum width. The size *s* of the perturbation decays with the distance *d* from the obstacle. See electronic supplementary material, appendix S1 of [4] for a derivation.

Similar reasoning applies to the case of hotspots. Figure 2*b* and electronic supplementary material, figure S2B display the front behind a circular hotspot, together with the paths back to the initial front. Most paths from the bulge to the initial front pass through the central region of the hotspot, implying that the hotspot length is important for front shape far behind the hotspot. Numerically, we find that two ellipses with equal length, but different width, result in very similar bulges of the front as shown in figure 3*b*.

We find the bulge to be heuristically well described by a radial wave originating at the hotspot’s centre and whose radius is given by

5.3 $$r=d+k,\;\mathrm{with}\;k=2l\left(1-\frac{v_{1}}{v_{2}}\right),$$

where *d* is the distance between the unperturbed front and the centre of the hotspot and *l* is the half-length of the hotspot (equal to radius for a circular hotspot). *v*_1_ and *v*_2_ are the front speeds surrounding and within the hotspot, respectively. This heuristic solution describes the bulge originating from a circular hotspot (figure 2*d*), elliptical hotspots (figure 3*b*), and even a tulip-shaped hotspot (electronic supplementary material, figure S4B) reasonably well. At its tip, the bulge proceeds the otherwise planar front by *k* defined above corresponding to the advance a virtual marker gains by passing through the hotspot along the axis of symmetry. Note that *k* does not depend on *d*, the distance travelled since the hotspot was encountered.

In the following, we will refer to the approximate solutions far away from the obstacle (the two emitted radial waves from the extreme borders) and hotspot (the emitted radial wave from the centre of the hotspot) as ‘far-distance solutions’ keeping in mind the heuristic nature for the case of hotspots.

Using the Eikonal equation (5.1), and the equations characterizing the far-distance solutions (5.2) and (5.3), one can illustrate an additional important property of the least-time description. If the environment including the obstacle or hotspot is stretched in all directions by the same factor, while front speed is kept constant, the arrival time is increased by the same factor, giving rise to a similarity solution of the front shape. This highlights that the findings presented can be applied at very different spatial scales, independent from the underlying mechanism of front propagation as long as the least-time principle can be applied. If this is not the case, for example if obstacles and hotspots are smaller than the characteristic front width discussed above, we expect a different front dynamics.

Taken together, we have seen how a least-time description of front propagation can predict the front computed with an individual-based simulation. This perspective allows us to characterize the perturbations induced by obstacles and hotspots, in particular the description as a superposition of the initial front with one or two radial waves, anticipated in figure 2*c*,*d* (dashed purple lines). In the following, we will use these findings to investigate the effect of multiple obstacles and hotspots on systems too large to be investigated by individual-based simulations.

## 6. Multiple obstacles and hotspots: a scattering process

How are the perturbations by single obstacles and hotspots affected by other features downstream? Or, conversely, how is the effect of a feature influenced by perturbations upstream? To answer these questions, we will first consider a dilute regime employing the findings for individual obstacles and hotspots before investigating the regime of a dense pattern of features.

**Figure 4.**
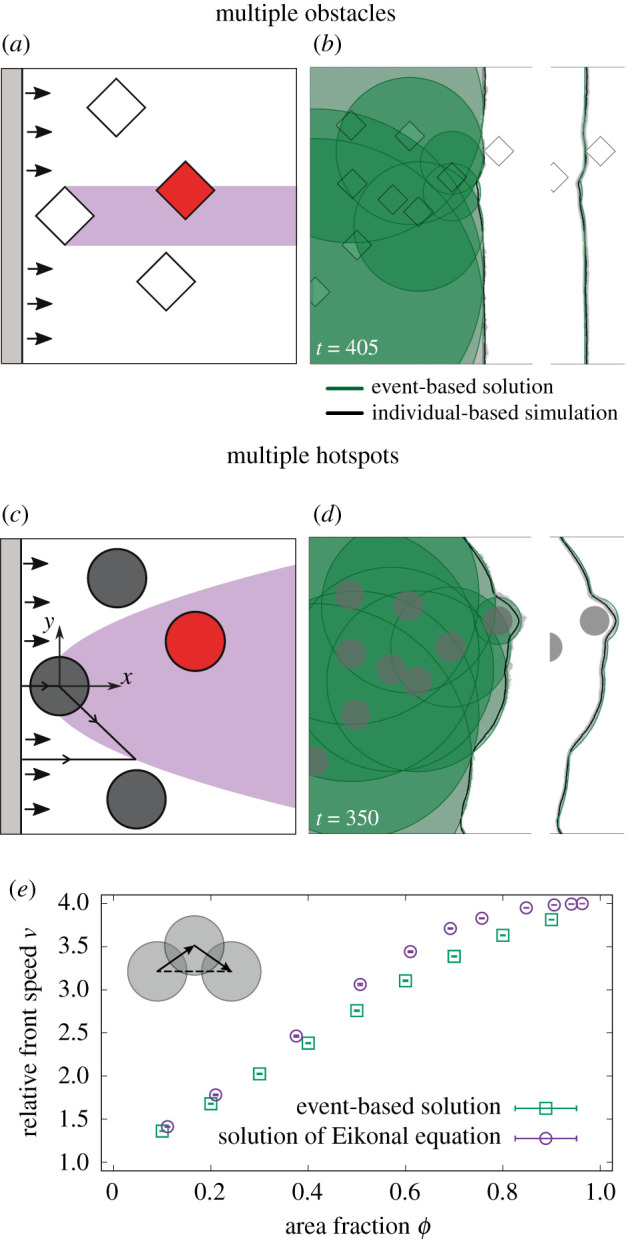
(*a*) Region within which an obstacle perturbs the front. The purple area illustrates the shadow of the first of four obstacles: obstacles inside this area (such as the red rhombus) will be affected by the perturbations created by the first obstacle. (*b*) Comparison of the event-based solution (green circles) with the result of individual-based simulations (grey dots for one realization) in the presence of rhombus-shaped obstacles (half-width *w* = 50). The envelope of the circles, determined by the event-based solution, matches well with the average front derived from many individual-based simulations (black line). (*c*,*d*) Similar to (*a*,*b*), but now for circular hotspots with radius *R* = 50 and *D*_2_ = 10 with radial waves originating from hotspots’ centres. (*e*) Speed-up of front *ν* (ratio of front speed in presence of hotspots and front speed outside of hotspots) computed with the event-based approach (green squares) and the numerical solution to the Eikonal equation (violet circles) for variable area fractions of hotspots *ϕ*. Inset: sketch of overlapping hotspots. The path through hotspots centres is longer than the shortest path between the leftmost and rightmost hotspot (dashed line).

Figure 4*a* displays four obstacles encountered by an original planar front. The purple region indicates the ‘shadow’ of the obstacle, i.e. the area influenced by the first obstacle encountered. Only the obstacle overlapping with this region, shown in red, interacts with the perturbation created upstream, causing a more complex perturbation, because the red obstacle is reached by a non-planar front. For rhombus-shaped obstacles considered here, the front in their shadow is completely described by the radial waves discussed above, i.e. each corner of a rhombus acts as a ‘scattering point’ from which a radial wave originates.

Front propagation in an environment with rhombus-shaped obstacles reduces to repeated scattering at the corners of rhombuses resulting in an ‘event-based solution’. The front is then constituted by the maximum (or envelope) of all radial waves (and the unperturbed planar front) which are not blocked by obstacles. Figure 4*b* illustrates the success of this approach: the black line indicates the average front derived from the microscopic individual-based model which agrees with the envelope of the green circles, the event-based solution, after a few rhombuses have been encountered by the front. While for rhombuses this scattering algorithm is exact, smooth curved boundaries would be associated with an infinite number of scattering events making this approach computationally unfeasible.

The perturbations induced by hotspots accumulate differently. The effect of a hotspot is not only felt in its geometrical shadow, but in a widening region as is evident from figure 3*b*. Using the heuristic approximation described above, the interaction region can be obtained by equating the distance *d* a planar front would travel after passing the hotspot’s centre with a radial wave of radius *d* + *k* with *k* defined in equation (5.3). The result is a sideways parabola, in the *x*–*y* reference system with origin at the centre of the hotspot,

6.1 $$y=\pm \sqrt{k^{2}+2kx}.$$

In figure 4*c*, the red hotspot, located within the parabola, will further accelerate the front, while hotspots indicated in dark grey are expected to advance the front independently.

The effect of several hotspots can be pictured as a succession of activation events: each hotspot encountered by the front is ‘activated’ and a radial wave originates from its centre. The planar wave and all radial waves can activate hotspots. The specific rules reflect that the front propagates with speed *v*_2_ inside and speed *v*_1_ outside the hotspot, respectively. The front is given by the envelope of all these individual circular waves and the initial planar front (see electronic supplementary material, appendix S1 for a detailed description). Figure 4*d* illustrates this approach (green circles) and shows good agreement with the front determined from the individual-based simulation (black line). Since the event-based algorithm for hotspots uses the heuristic solution for large distances, we expect the resulting front to generally deviate from the exact solution.

Front speed is a key observable for the spatial spread of populations and the observable focused on below. We therefore use front speed to quantify the deviation between the event-based solution and the exact solution obtained by solving the Eikonal equation numerically, introduced above and described in electronic supplementary material, appendix S1. Front speed is derived from the (mean) front position, defined as front position averaged along the direction vertical to the advancing front (averaged in vertical direction in figure 4) and reported relative to front speed in the absence of obstacles or hotspots, i.e. relative to *v*_1_.

Figure 4*e* displays this relative front speed *ν* in the presence of random hotspot configurations at variable area fractions *ϕ*, i.e. different fractions of area covered by hotspots. Front speed derived from the event-based solution appears in good agreement with that from solving the Eikonal equation for small hotspot area fractions of up to *ϕ* ≈ 0.3. For intermediate area fractions of *ϕ* ≈ 0.6, front speeds obtained with both approaches deviate from each other significantly. At very high area fractions, both approaches result in an effective speed close to the speed expected in an environment fully covered with hotspots (*ν* = 4 for *v*_2_/*v*_1_ = 4). In general, the event-based approach underestimates front speed because in the event-based solution only paths through hotspot centres are considered even though shorter paths may exist (inset to figure 4*e*). This effect plays a minor role in the dilute regime and in the regime of very dense hotspots. In the former case, we expect the heuristic solution to describe the front well. In the latter case many, potentially aligned, hotspots exist. Taken together, the event-based solution provides qualitative insight into front dynamics; it is not suited to compute front speed for general inhomogeneous environments.

Having established that the least-time principle can be used to describe front dynamics around isolated and small groups of inhomogeneities, we will use the numerical solutions to the Eikonal equation to explore the front dynamics for much larger systems, different shapes of obstacles and hotspots, and for a wide range of area fractions. While it is not necessary to run the individual-based simulations to expanding populations in those environments, it would also be prohibitively costly computationally.

## 7. Front speed as function of obstacle density and shape

The picture of individual obstacles inducing scattering events leads to a number of predictions: several obstacles located in each others’ shadows perturb the front repeatedly and, if occurring at all parts of the front simultaneously, lead to an overall slow-down of the front. Since perturbations originating from single obstacles heal with increasing distance from the obstacle, the cumulative effect of perturbations becomes stronger if obstacles are closer, i.e. in a denser configuration.

To test these predictions, we numerically solved the Eikonal equation using the fast marching method for elliptical obstacles in a system as large as computationally feasible. We investigated elliptical obstacles such as in figure 3*a* because they are arguably less idiosyncratic then rhombuses. Without loss of generality, we chose the spatial scale of obstacles to be of order 1. With the lattice constant chosen to be 1/15, each obstacle is represented by hundreds of lattice sites. The width of the channel with periodic boundary conditions is set to 50 and the length to 1300; see electronic supplementary material, appendix S1 for more details. We computed the front dynamics for a random placement of obstacles at a given number density *ρ* and obtained the front speed by linear fits of front position versus time as described in electronic supplementary material, appendix S1.

**Figure 5.**
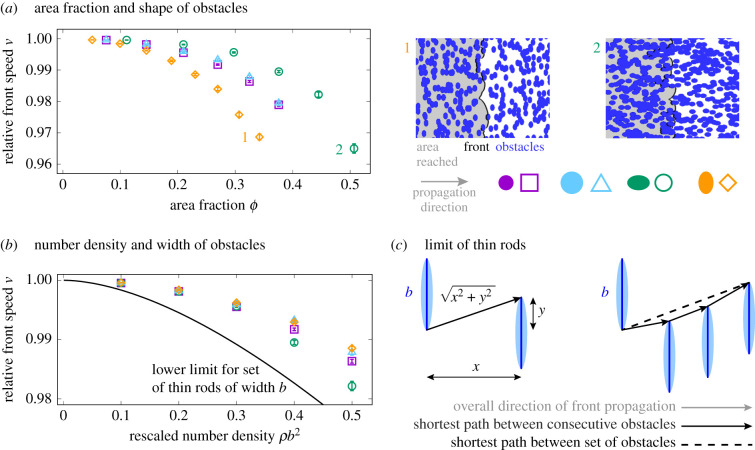
Effect of many randomly placed obstacles on front speed. (*a*) (Left) Slow-down of front quantified by relative front speed *ν* obtained by numerically solving the Eikonal equation as a function of *ϕ*, the area fraction covered by obstacles. Symbols and colours indicate ellipse-shaped obstacles with varying aspect ratio (purple and blue: 1, green: 3/2, orange: 2/3). (Right) Snapshots of obstacle configurations and resulting front shape from the numerical solution for parameters indicated on left. See electronic supplementary material, videos S3 and S4 for corresponding videos of front movement. (*b*) Relative front speed *ν* for elliptical obstacles defined and computed as in (*a*), but as function of *ρb*^2^, with *ρ* the number density of obstacles and *b* the obstacle width. In addition, a lower limit for front speed in the presence of a system of thin rods is shown (black line, see electronic supplementary material, appendix S2). (*c*) (Left) A sketch of the shortest path between the two rods or very elongated ellipses of width *b*, distance *x*, and overlap *y*. The relative increase in path length is given by $\sqrt{x^{2}+y^{2}}/x$. (Right) A sketch of possible paths through a geometry with randomly distributed parallel rods. The dashed line shows the absolute shortest paths, the solid arrows show the path constructed from the shortest paths between consecutive obstacles.

Figure 5*a* displays relative front speed *ν* as a function of area fraction *ϕ* for four different ellipses which differ in length and width as well as snapshots of obstacle configurations and resulting front shape. Area fraction is a function of the product of number density *ρ* and the semimajor and semiminor axes *R*_*a*_ and *R*_*b*_, i.e. *ϕ* = 1 − exp(−*ρπR*_*a*_*R*_*b*_) as easily derived by change of variables and the well-known result for overlapping discs [32]. As expected, for circular obstacles (purple and cyan symbols), front speed does not depend on the radius because the obstacle and front shape can be scaled with the same factor, as discussed above. At equal area fraction and for ellipses with same aspect ratio (green and orange symbols) front speed is more reduced if the long axis is parallel to the front, in agreement with our finding that it is the obstacle’s cross section that is responsible for the front perturbation. However, this observation also implies that for a given environment, with aligned obstacles, front speed can depend on the angle of incidence and the environment can therefore be anisotropic with respect to front propagation.

We had seen that the ‘far-distance solution’ of the front behind an obstacle is characterized by the obstacle’s width *b* (but not its shape). Because the speed does not change when stretching the environment in all dimensions equally, speed cannot depend on *b* alone or on non-dimensionless combinations of *b* and the number density *ρ*, but instead should be a function of the dimensionless parameter *ρb*^2^. We indeed observe a strong dependency of speed *ν* on *ρb*^2^ (figure 5*b*). However, this dependency is imperfect: the front is slower for larger length to width ratios (i.e. for ‘longer’ ellipses). This is because for ‘longer’ ellipses, a larger fraction of the area is covered by obstacles increasing the path length. Conversely, a (hypothetical) system of thin rod-shaped obstacles is expected to provide an *upper limit for front speed* in an environment of obstacles with width *b* and number density *ρ*.

A system of very thin rods lends itself to an understanding of the cause for the slow-down. When the projections of rods in the direction of front propagation overlap, the propagation path will graze the corners of the rods (similar to the ‘scattering description’ we employed above). The slow-down of the front is then given by the increase in path length, relative to the straight path in the propagation direction, as shown in figure 5*c*. The path grazing the corners of all overlapping rods is however not always the shortest path as evident in the exemplary configuration in figure 5*c*. There, the shortest path directly connects the first and the last rod (dashed line), while considering nearest neighbours (solid arrows) constructs a longer path that connects all rods in between. Assuming the path grazes all consecutive overlapping rods allows one to compute front speed analytically by integrating over all possible overlapping rod pairs (see electronic supplementary material, appendix S2). The result (black line in figure 5*a*) is a *lower limit for front speed* for a system of thin rods.

Taken together, we derived a lower limit for front speed in a system of rods of length *b* which itself sets an upper limit for sets of obstacles of width *b*. This finding is nevertheless useful as it sheds light on why obstacles reduce front speed only marginally. It is not the area covered by obstacles that sets front speed, but the extension of path length that is required to graze the corners of (a subset of) obstacles.

However, with increasing density, the shape of the obstacles becomes important and obstacles may overlap more often. Since the front cannot propagate inside obstacles, the front will stop when so many obstacles overlap in transversal direction that no unobstructed path exists. Such blockages can arise in finite domains even at a filling fraction smaller than the critical percolation threshold, which is for circular obstacles in an infinite system given by *ϕ* ≈ 0.68 [32,33]. We have limited our analysis to significantly lower area fractions, for which statistics on the front speed can still be easily acquired. We expect the front to slow down dramatically close to or above the percolation threshold. This slow-down has been addressed recently in lattice-based growth models [20,34].

## 8. Front speed as function of hotspot density, shape and intensity

**Figure 6.**
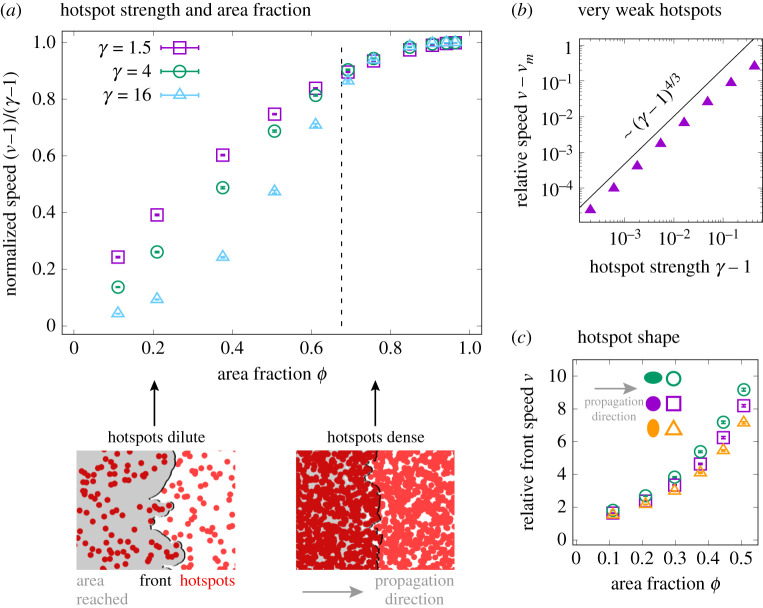
Effect of many randomly placed hotspots on front speed, obtained by numerically solving the Eikonal equation. (*a*) (Top) Normalized front speed (*ν* − 1)/(*γ* − 1) as function of hotspot strength *γ* and area fraction *ϕ*. The dashed vertical line corresponds to the percolation threshold in an infinite system (*ϕ* ≈ 0.68). See electronic supplementary material, figure S5 for the same numerical results, plotted relative to the weighted harmonic mean of local front speeds. (Bottom) Snapshots of dilute and dense hotspot configurations and resulting front shape. See electronic supplementary material, videos S5 and S6 for corresponding videos of front movement. (*b*) Relative speed *ν* − *ν*_*m*_ with *ν*_*m*_ = *ϕγ* + (1 − *ϕ*), the spatial average of local front speed. The solid line indicates a power law ∼(*γ* − 1)^4/3^. (*c*) Relative front speed *ν* as a function of area fraction *ϕ* for strong elliptical hotspots with strength *γ* = 16 for three different aspect ratios (purple: 1, green: 3/2, orange: 2/3). See electronic supplementary material, figure S6 for an equivalent plot, but for weak hotspots with *γ* = 1.5.

A single hotspot leads to a transient increase in local front speed, resulting in a bulge with constant size in the direction of front movement and sideways spreading along the front (figure 3*b*). We therefore expect multiple hotspots to result in an overall speed-up of the population front. We first consider the case of circular hotspots with intensity *γ* = *v*_2_/*v*_1_ and area fraction *ϕ*. Figure 6*a* depicts the speed-up as obtained from solving the Eikonal equation. Relative front speed *ν* is plotted as (*ν* − 1)/(*γ* − 1), which varies between 0 and 1 for any *γ* and any *ϕ* between 0 and 1.

The shape of the speed-up *ν*(*ϕ*) depends on hotspot strength *γ*. While for small *γ*, it resembles a concave function, we observe a sigmoidal shape for large *γ* with the point of inflection at an intermediate area fraction below the percolation threshold (*ϕ* ≈ 0.68 for an infinite system). We hypothesize that the larger slope at intermediate *ϕ* is due to a change in how the front is sped up when increasing *ϕ*: while for a dilute system, the front is locally accelerated by individual hotspots (figure 6*a*; electronic supplementary material, video S5), for large area fractions the hotspots constitute a connected path and the effective front speed depends on the length of this percolating path (figure 6*a*; electronic supplementary material, video S6). In a finite domain, percolation can occur below or above the percolation threshold in the thermodynamic limit, depending on the actual hotspot configuration. We expect this fact to be reflected in a larger variance in the measured speed, 〈(*ν* − 〈*ν*〉)^2^〉, close to the critical area fraction.

In a simple linear habitat, as sketched in figure 1*b*, the front speed along this linear path does not depend on the arrangement of hotspots, but solely on the area fraction *ϕ*. The relative front speed, *ν*, is given by the weighted harmonic mean, *ν*_*h*_ = (*ϕ*/*γ* + (1 − *ϕ*))^−1^ (electronic supplementary material, appendix S2). This result is a lower bound for the front speed-up in two dimensional systems, since in the latter many more paths with possibly shorter travel times exist, in addition to a straight path mimicking a linear habitat. Indeed, (*ν* − 1)/(*ν*_*h*_ − 1), depicted in electronic supplementary material, figure S5, is larger than one for all area fractions. It is largest around the percolation threshold and for large hotspot strength.

For *γ* ≈ 1, i.e. very weak hotspots, the results from scaling [35], numerical [36], and mathematical analysis [37] of the speed-up of a Huygens front in isotropic random media apply to our system. In particular, we expect the speed-up minus the relative spatial average of local front speed, *ν*_*m*_ = *ϕγ* + (1 − *ϕ*), to scale with the strength of the perturbation, *γ* − 1, as

8.1 $$\nu -\nu_{m}\propto {\left(\gamma -1\right)}^{4/3}.$$

Figure 6*b* is consistent with this prediction for *ϕ* = 0.5. So far, we have considered circular hotspots and addressed the dependence of the speed-up on their intensity and area fraction. As discussed above, the length of an individual hotspot determines much of the front shape downstream. In particular, ellipses with equal length but different aspect ratio result in very similar front shapes (figure 3*b*). Conversely, we expect that ensembles of longer hotspots speed up the front more than ensembles of wider hotspots at equal area fraction. Numerical solutions confirm these predictions; see figure 6*c* for strong hotspots and electronic supplementary material, figure S6 for weak hotspots of varying aspect ratio.

## 9. Discussion

The effect of inhomogeneities on population fronts depends on the type of inhomogeneities perturbing the front. Both classes of features considered here, obstacles and hotspots, perturb the population front in their own distinct way. The kink caused by an obstacle is transient and limited to the obstacle’s width. Hotspots create a permanent perturbation that spreads along the front. Both effects can readily be understood by least-time arguments and analogies to geometrical optics at sufficiently large scales. Far from the inhomogeneity, the front can be described as a combination of radial waves induced from the outer corners of an obstacle or from the centre of a hotspot, respectively, which paints a picture of front propagation by repeated scattering events in environments with many inhomogeneities. On the quantitative side, the front speed can be obtained numerically using the fast marching method, i.e. by solving the Eikonal equation. This allowed us to investigate dependence of front speed on the environment’s parameters such as area fraction of the features’ shape.

**Figure 7.**
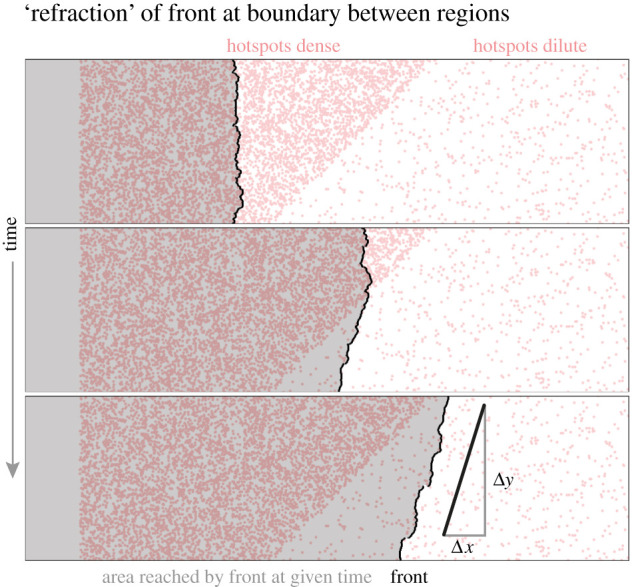
‘Refraction’ of a front, obtained by numerically solving the Eikonal equation, at an interface between a region with dense (*ρ* = 0.150) and dilute hotspots (*ρ* = 0.015) of strength *γ* = 2.0, tilted at 45° relative to the initial front. Upon encountering the interface, the front changes overall direction, manifesting in a tilt. The tilt angle is in agreement with the prediction based on the measured front speed at the area fractions of hotspots to the left and right using Snell’s Law [26] (*v*_left_ = 1.74, *v*_right_ = 1.14, $\Delta y/\Delta x=\tan(\pi /4+\arcsin(v_{\mathrm{right}}/\sqrt{2}v_{\mathrm{left}})))$. We attribute deviations to boundary effects at the top and bottom of the channel. See electronic supplementary material, video S7 for a video of the full solution.

The least-time description and the Eikonal equation employed here also arise in geometrical optics. Intuition gained from studying optics carries over to a large extent. To push the analogy further onto larger length scales, let us consider two areas with different hotspot density placed next to each other, with the interface tilted by 45° with respect to the initial front direction as illustrated in figure 7. From figure 6*a*, we expect the front to propagate faster at high than at low hotspot density—and thus refraction of the front at the interface. Indeed, figure 7 illustrates that as the front transitions from the region with dense hotspots to the region with dilute hotspots, it changes overall direction. The refraction angle predicted from Snell’s Law with propagation speeds measured in analogous homogeneous systems matches the observed tilt of the front.

The analogy to geometrical optics arises whenever fronts propagate in normal direction with a locally determined speed that is independent from, for example, front curvature, and thus found its application in other fields such as the prediction of forest fire fronts [38]. However, the analogy with optical phenomena is limited. For example, constructive and destructive interference will not occur in population expansions considered here. Reflection, which can be derived from Fermat’s principle of least time [26], cannot be observed, because populations always expand into empty domains.

A large body of literature has investigated the effects of heterogeneities in one-dimensional, in particular periodic, habitats (e.g. [5–7]). Our study highlights that the results for linear habitats are generally not transferable to higher dimensions and thus not to many scenarios in nature. In the case of obstacles embedded in a two-dimensional environment, stagnation of front propagation can only occur when the area fraction is around or above the percolation threshold and there is no ‘free path’ available to propagate further. In the case of hotspots, propagation is faster than in a corresponding linear habitat since many more paths are available. Thus, two-dimensionality suppresses the effect of obstacles and intensifies the effect of hotspots. We limited ourselves to random ensembles of potentially overlapping features of equal shape and orientation. Numerous questions arise that may be topics for future research. (i) A system of dense hotspots can be interpreted as a system of dilute imperfect obstacles, which are not circular. Can we predict front speed in this regime building on the statistics of the complement of overlapping discs [32]? (ii) If obstacles are placed such that open channels exist within which the front can propagate undisturbed, front speed is not affected. Can we better predict front speed by identifying these channels? (iii) What is the front dynamics in complementary environments such as those generated from fractals [22]?

We envision our findings to support the study of macroscopic invasions in two different ways. Firstly, if researchers find evidence that a population expansion is governed by spread in normal direction, they can follow our approach of numerically solving the Eikonal equation to make predictions for front position at later times. This is especially useful should they wish to predict the front in large systems or for a large number of different habitats, which is not feasible with individual-based simulations. Secondly, we believe the intuition gained from geometrical arguments can be used to understand even those environments which do not fulfil the requirement of a local front speed.

The least-time considerations and the Eikonal equation are fully deterministic and cannot capture fluctuations present in a single realization of a population front of discrete individuals such as illustrated in figure 2. While we found the average over many realizations to be well described by the deterministic least-time consideration, it is possible that fluctuations drive individual expansions into different overall front dynamics, a question that warrants further investigation. Relatedly, deterministic dynamics of the population front does not imply deterministic evolution of the expanding populations. Even if the population expands its range mostly deterministically, a small population size at the front and the associated large genetic drift lead to gene surfing and gene segregation [39,40]. The evolutionary dynamics is thereby influenced by the shape and dynamics of the front [40,41]. Previous work has shown how the effects of obstacles and bumps on the evolutionary dynamics can be understood using the dynamics of front shape [4,19]. In particular, there is a relationship between lineages, the set of locations of subsequent birth events, and the shortest path used to construct the front in the analytical solution [19]. This suggests that this work, in particular the characterization of paths of least time, might help understand the collective effect of many large obstacles or hotspots on the genetic composition of the invading population, complementing recent work that characterized lineages in disordered environments without spatial correlation [20].

## Data accessibility

The source code corresponding to the event-based solution and the solution of the Eikonal equation is available on Zenodo, https://doi.org/10.5281/zenodo.5513567, and GitHub, https://github.com/wmoebius/inhomogeneities_one2many.
